# Neurotransmitter co-transmission: synaptic architectures, functional logic, and emerging tools

**DOI:** 10.3389/fnmol.2026.1862021

**Published:** 2026-06-18

**Authors:** Elif Tunc-Ozcan

**Affiliations:** Department of Neurosciences, University of New Mexico, Albuquerque, NM, United States

**Keywords:** neurotransmission, neurotransmitter co-release, neurotransmitter co-transmission, synaptic architectures, synaptic plasticity

## Abstract

Neurotransmitter co-transmission has become recognized as a fundamental organizing principle of neural communication, challenging the traditional view that individual neurons operate through a single transmitter system. Current evidence demonstrates that many neurons utilize multiple transmitters via distinct synaptic architectures, such as co-packaging within the same vesicle, release from separate vesicle pools within the same terminal, and segregation of transmitters across different boutons or neuronal processes. These organizational modes are not simply structural variants; they impose distinct rules for release, target engagement, and short-term dynamics, thereby shaping circuit function in specific ways. Across neural systems, several common principles have emerged: co-transmission expands signaling across multiple timescales, enhances target specificity, and allows transmitter balance to shift according to firing patterns and circuit state. A major conceptual and technical challenge in the field is that no single method can definitively establish the release mechanism. Consequently, recent advances have relied on integrating molecular profiling, electrophysiology, high-resolution anatomy, optogenetics, and genetically encoded neurotransmitter sensors. Collectively, these approaches are beginning to clarify how multi-transmitter neurons are organized and how their signaling is regulated. Future progress will likely depend on multimodal strategies that connect synaptic architecture to release dynamics, circuit computation, and behavior *in vivo*. In this context, co-transmission should be viewed not as an exception to canonical neurotransmission but as a versatile mechanism that enhances the flexibility, precision, and context-dependence of neural circuit output.

## 1 Introduction

For decades, transmitter identity has functioned as a proxy for neuronal identity, such as glutamatergic neurons excite, GABAergic neurons inhibit, cholinergic neurons modulate, and monoaminergic neurons broadcast slow signals. Although this framework remains useful, accumulating evidence shows that many neurons possess the molecular machinery for more than one transmitter and can use multiple transmitters to influence the same or different targets (Wallace and Sabatini, 2023). Co-transmission is therefore increasingly viewed not as an exception, but as a broader organizational principle of neural circuits (Granger et al., 2017). At the same time, the field still lacks a clear understanding of how co-transmission is regulated and how it shapes circuit function under physiological and pathological conditions.

**Figure 1:**
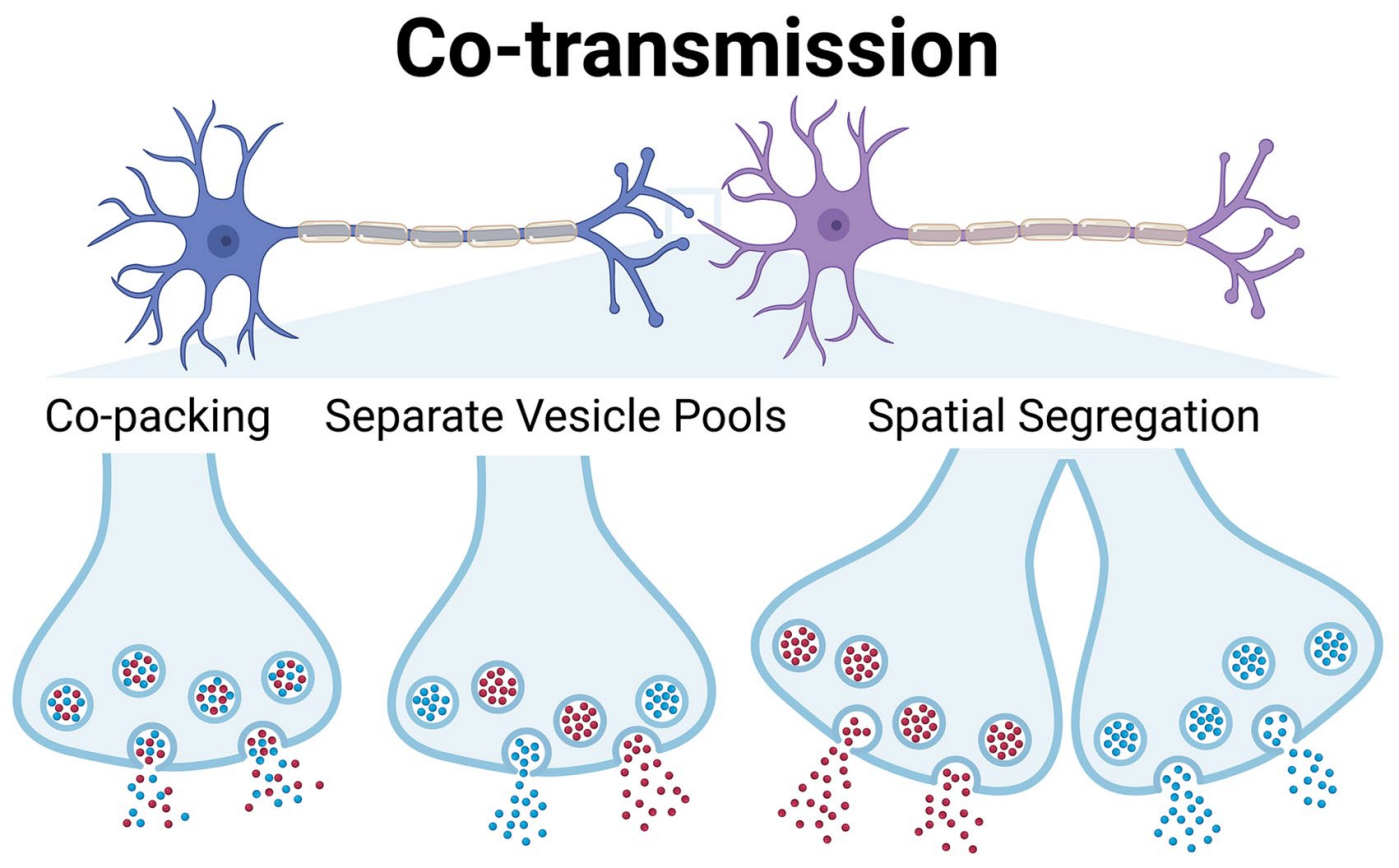
Schematic demonstration of the co-transmission.

A major challenge has been inconsistency in terminology. Co-expression refers to the presence of molecular markers from multiple transmitter systems, such as synthetic enzymes or vesicular transporters, within a single neuron; however, this observation alone does not confirm synaptic release (Mazon et al., 2026). Co-transmission is the broader functional concept, indicating that a neuron uses more than one transmitter to influence downstream targets (Takacs et al., 2018). Co-release usually refers to the release of multiple transmitters from the same terminal during a single signaling event, whereas co-packaging makes the stronger mechanistic claim that multiple transmitters are contained within the same synaptic vesicle and therefore released together (Vaaga et al., 2014; Tritsch et al., 2016). Distinguishing among these terms is essential for interpreting studies of multi-transmitter neurons and for avoiding conclusions that extend beyond the available evidence (Figure 1).

## 2 Organizational architectures of co-transmission

Once co-transmission is defined conceptually, the next question is how it is organized at the synaptic level. Available evidence indicates that co-transmission can be implemented through multiple distinct release architectures. One prominent model is same-vesicle co-packaging, where two neurotransmitters are loaded into a single synaptic vesicle and released simultaneously. Early functional support for this model came from spinal inhibitory synapses, where Jonas and colleagues showed that interneuron-motoneuron synapses corelease glycine and GABA. Because a subset of miniature IPSCs contained both glycinergic and GABAergic components, their findings support co-packaging and quantal corelease of glycine and GABA from the same synaptic vesicle (Jonas et al., 1998). Additional support for same-vesicle co-packaging came from habenular cholinergic projections to the interpeduncular nucleus, where Ren and colleagues showed that “cholinergic” terminals co-release glutamate and acetylcholine. They found that many terminals contain both the vesicular glutamate transporter and the vesicular acetylcholine transporter within the same synaptic vesicle fraction (Ren et al., 2011). Another well-characterized example involves entopeduncular projections to the lateral habenula, in which glutamate and GABA are released from the same pathway (Kim et al., 2022). Although molecular and ultrastructural studies had confirmed that these terminals possess the necessary machinery for both transmitter systems, they did not conclusively determine whether the two transmitters are released from the same vesicle or from separate vesicle pools. Kim and colleagues addressed this issue by comparing co-packaging and independent release models using patterned optogenetics, electrophysiology, and statistical modeling (Kim et al., 2022). Their findings indicate that many somatostatin-positive entopeduncular terminals in the lateral habenula exhibit properties most consistent with glutamate-GABA co-packaging, although some sites support independent release. Collectively, these results suggest that co-transmission is not governed by a single universal mechanism, even within a single projection, and that synaptic organization is a critical determinant of how multi-transmitter signaling influences circuit function.

A second organizational mode involves the release of neurotransmitters from separate vesicle pools within the same presynaptic terminal. In this configuration, two transmitters originate from the same bouton but are packaged into distinct vesicles, enabling differential release properties. This organization is illustrated by starburst amacrine cells in the retina, which co-release acetylcholine and GABA onto direction-selective ganglion cells (Lee et al., 2010). Lee and colleagues showed that acetylcholine and GABA release differ in calcium dependence and short-term plasticity, supporting the conclusion that these transmitters are released from separate vesicle populations rather than being co-packaged in the same vesicles (Lee et al., 2010). Functionally, this arrangement allows acetylcholine to provide motion-sensitive excitation, while GABA provides directionally biased inhibition that sharpens direction selectivity. Recent studies on supramammillary projections to dentate granule cells provide another example of this model (Hirai et al., 2024). Hirai and colleagues demonstrated that, although supramammillary terminals co-transmit glutamate and GABA, the two signals differ in paired-pulse behavior, calcium sensitivity, presynaptic receptor modulation, and short-term plasticity (Hirai et al., 2024). Minimal stimulation and asynchronous release experiments revealed predominantly independent excitatory and inhibitory quantal events, while anatomical analyses showed that individual terminals form closely apposed yet distinct glutamatergic and GABAergic postsynaptic specializations. Collectively, these findings support the conclusion that glutamate and GABA are released from separate vesicle populations within the same axons, and that this arrangement enables frequency-dependent filtering of supramammillary input to the dentate gyrus.

A third organizational mode is neurotransmitter segregation, in which different transmitters are directed to distinct terminals or neuronal processes rather than being released from the same bouton. In this configuration, a single neuron can deliver different chemical signals to separate targets, resulting in variable transmitter output across its arbor. Cifuentes and Morales characterize segregation as a plastic subtype of co-transmission regulated by synaptic demands and target-derived signals, highlighting examples from both central and peripheral circuits where segregation enables transmitters to act on separate postsynaptic compartments or distinct target cells (Cifuentes and Morales, 2021). Consistent with this framework, Fortin et al. demonstrated that dopamine neurons can establish largely distinct dopamine- and glutamate-releasing varicosities, often along the same axon, and further showed that contact with striatal target cells influences this organization: ventral striatal neurons promoted segregation of release sites, whereas dorsal striatal neurons suppressed the glutamatergic phenotype by reducing VGLUT2 expression (Fortin et al., 2019). Similar target-dependent heterogeneity is seen in dorsal raphe serotonergic projections. Gagnon and Parent showed that individual dorsal raphe axons are highly collateralized and contain both VGLUT3-positive and VGLUT3-negative serotonergic varicosities along the same axonal segments, with a higher proportion of VGLUT3-positive boutons in the striatum than in motor cortex (Gagnon and Parent, 2014). These findings suggest that a single serotonergic neuron may release serotonin alone at some boutons while retaining the machinery for serotonin-glutamate co-transmission at others, depending on projection target and local bouton identity. These findings are important because they avoid the assumption that all terminals of a multi-transmitter neuron operate identically and instead emphasize that target-dependent transmitter organization can diversify circuit signaling and functional output.

## 3 Functional logic of co-transmission

Beyond synaptic organization, co-transmission expands circuit function through several recurring principles. The first is temporal layering. By integrating fast ionotropic signals with slower metabotropic or modulatory actions, co-transmission enables a single pathway to influence a circuit across multiple timescales. This principle is particularly evident in small-molecule–peptide systems, where rapid synaptic effects are followed by longer-lasting changes in excitability, synaptic strength, or network configuration (Nusbaum et al., 2017). Nusbaum and colleagues provide numerous examples in which co-transmitters act either convergently on the same target or in parallel on different elements of a microcircuit, thereby increasing both the flexibility and robustness of circuit output (Nusbaum et al., 2017). For example, in oxytocin neurons, low concentrations of oxytocin enhance GABAergic synaptic transmission through a presynaptic mechanism. These inhibitory inputs subsequently trigger post-inhibitory rebound firing via low-threshold calcium channels, ultimately strengthening bursting activity (Israel et al., 2008). This example is important because it shows that co-transmission does not simply add another signal to a circuit; it can reorganize the timing and mode of recruitment of network activity, allowing slow peptide signaling to reshape the functional consequences of fast synaptic inhibition.

A second principle is target specificity. Co-transmission does not necessarily produce identical signals at every synapse formed by a neuron. When transmitters are segregated across boutons, branches, or postsynaptic specializations, a single neuron can communicate differently with distinct targets. This principle is exemplified by neurotransmitter segregation, in which transmitters are routed to specific neuronal processes or terminals, enabling one axonal arbor to deliver different chemical messages to various cells or subcellular compartments. Cifuentes and Morales emphasize that this organization is functionally significant because it expands the signaling repertoire of individual neurons and cautions against assuming uniform transmitter identity across all terminals of a multi-transmitter cell (Cifuentes and Morales, 2021).

Cai and Ford provide a clear example of target- and region-specific co-transmission within a single broad projection system (Cai and Ford, 2018). They showed that activation of substantia nigra pars compacta dopamine neurons has opposing effects on striatal cholinergic interneurons depending on the postsynaptic region. In the dorsomedial striatum, dopamine neuron activation suppresses cholinergic interneuron firing through D2 receptor signaling, whereas in the dorsolateral striatum, the same projection drives cholinergic interneuron bursting through glutamate co-release and activation of group I metabotropic glutamate receptors. Together, these findings show that dopamine-glutamate co-transmission helps balance regional differences in cholinergic signaling across the dorsal striatum. This study illustrates that co-transmission is not simply redundant; instead, the relative contribution of each transmitter can vary across projection targets to produce distinct circuit-level effects.

A third principle is state dependence. The impact of co-transmission depends not only on the transmitters present but also on the mechanisms governing their release. Distinct vesicle pools can exhibit different calcium sensitivities, coupling arrangements, presynaptic modulation, and activity dependence, allowing the balance of transmitter release to shift with firing patterns or circuit state. Takács and colleagues illustrate this principle in septo-hippocampal cholinergic terminals, where acetylcholine and GABA are released from separate vesicle populations, are controlled by different voltage-dependent calcium channels, and are further cross-regulated by presynaptic muscarinic and GABAB autoreceptors (Takacs et al., 2018). Functionally, the GABAergic component exhibits strong short-term depression, whereas the cholinergic component is maintained more effectively across stimulus trains, indicating that the relative contribution of each transmitter changes with activity. Using midbrain dopamine neurons as the model, Silm and colleagues also show that dopamine neurons can release glutamate and dopamine with different synaptic properties, indicating that the two transmitters are segregated into different vesicle populations (Silm et al., 2019).

The striatal dopamine-GABA system illustrates this principle particularly clearly. Zych and Ford showed that both dopamine and GABA depend on VMAT2 for vesicular loading, but their release is not regulated identically (Zych and Ford, 2022). Instead, dopamine and GABA show different calcium sensitivity, release probability, active-zone protein dependence, and presynaptic neuromodulation, suggesting that even transmitters loaded through the same vesicular transporter can be functionally separated at the synaptic level. The authors also found that presynaptic neuromodulators regulate the two transmitters differently. Activation of D2 autoreceptors, GABAB receptors, and κ-opioid receptors inhibited dopamine release more strongly than GABA release. This indicates that dopamine and GABA co-transmission can be independently tuned by presynaptic receptor systems, even though both transmitters are released from the same broad class of axon terminals. This asymmetric neuromodulation means that the chemical output of a single projection can be reweighted by local receptor engagement: under one modulatory state, dopamine signaling may be preferentially suppressed while GABAergic inhibition is relatively preserved. Such independent regulation provides a mechanism by which co-transmitting neurons can shift circuit output without changing firing rate alone. These findings broadly support the concept that co-transmission is dynamically regulated by presynaptic mechanisms and circuit state, enabling the same projection to generate different network effects under varying conditions.

A fourth, closely related principle is temporal computation at the postsynaptic membrane. Krubitski and colleagues used compartmental and network modeling to show that glutamate-GABA co-transmission cannot be reduced to a simple algebraic sum of excitation and inhibition (Krubitski et al., 2026). Instead, the postsynaptic effect depends on the relative timing, amplitude, and kinetics of AMPA- and GABAA receptor-mediated components. When excitatory and inhibitory signals overlap with different rise times or latencies, the same co-transmitting synapse can generate prolonged excitation, net inhibition, or a brief transient biphasic response. This finding is conceptually important because it suggests that co-transmission can encode temporal information through the shape of the compound postsynaptic response, not only through the presence or absence of each transmitter.

Ceballos et al. provide experimental support for this temporal framework by showing that glutamate and GABA can be released in tightly linked miniature synaptic events across multiple brain regions (Ceballos et al., 2024). In striatal neurons, they detected biphasic minis in which AMPA and GABAA receptor-mediated components occurred together more often than expected by chance. These events appeared in both “straight” and “reverse” forms, indicating that excitation can precede inhibition or inhibition can precede excitation at the millisecond scale. This study shows that coordinated release of the two transmitters can generate temporally structured postsynaptic responses. Functionally, such timing differences could allow the same co-releasing input to produce rapid excitation followed by local inhibition, or a brief inhibitory/shunting phase followed by excitation, thereby shaping postsynaptic integration with high temporal precision.

## 4 Implications for circuit function and adaptation

The functional consequences of co-transmission are most effectively analyzed at the circuit level, where multiple neurotransmitters can alter both the strength of synaptic connections and the computational functions performed by specific pathways. In excitatory-inhibitory co-transmitting systems, a single projection may deliver temporally coordinated excitation and inhibition, generate feed-forward inhibition, or refine the temporal window for postsynaptic integration. In modulatory co-transmitting systems, a fast-acting transmitter can transmit temporally precise signals, while a slower co-transmitter modulates excitability or synaptic plasticity. Consequently, co-transmission enables a single anatomically defined pathway to function as multiple distinct channels.

This computational framework elucidates the significance of the anatomical location of co-transmission. According to the Krubitski et al. model, somatic and dendritic glutamate-GABA co-transmission result in distinct summation regimes due to the influence of dendritic filtering, soma-dendrite coupling, and active conductances on the transfer of compound signals to the soma (Krubitski et al., 2026). Persistent sodium conductance enhances excitatory summation, while hyperpolarization-activated conductance modifies temporal summation and promotes selective responsiveness to input frequency. Therefore, co-transmission interacts with the intrinsic electrical properties of the postsynaptic neuron to generate input filters that are sensitive to synapse location and stimulation frequency. This consideration is particularly relevant for understanding neural adaptation. Variations in co-transmitter balance, influenced by factors such as firing frequency, calcium source, autoreceptor engagement, and target identity, enable multi-transmitter neurons to modify their output without altering overall connectivity. Thus, co-transmission provides a mechanism for circuit flexibility, allowing the same axons and postsynaptic targets to persist while the chemical composition of the signal adapts to physiological context.

These characteristics have significant implications for disease and maladaptive plasticity. Alterations in the relative expression or release probability of co-transmitters, resulting from experience, stress, drug exposure, or developmental perturbation, may not simply increase or decrease circuit activity but can shift the balance among excitation, inhibition, and neuromodulation. Such shifts may affect oscillatory states, behavioral salience, sensory gain, reward valuation, or cognitive flexibility, depending on the specific circuit involved. Therefore, research on co-transmission should extend beyond identifying neurons with multiple transmitters to investigating how the relative timing, target distribution, and state-dependent recruitment of each transmitter influence circuit output and behavioral adaptation.

## 5 Methodological limitations and emerging tools

A central challenge in the co-transmission field is that no single method can independently establish the mechanism of neurotransmitter release. While molecular co-expression of synthetic enzymes, vesicular transporters, or transmitter-related transcripts can identify candidate multi-transmitter neurons, these approaches do not demonstrate synaptic release, distinguish co-packaging from parallel vesicle pools, or exclude segregation across terminals. Similarly, compound postsynaptic responses may indicate that a single projection engages multiple transmitter systems, but conventional stimulation typically aggregates responses from many boutons, potentially obscuring synapse-to-synapse heterogeneity. Consequently, recent reviews have emphasized that robust mechanistic conclusions require convergent molecular, physiological, and anatomical evidence, rather than inference from a single dataset (Wallace and Sabatini, 2023; Tritsch et al., 2016; Nusbaum et al., 2017).

Recent studies demonstrate progress toward meeting this evidentiary standard. For example, Hashimotodani and colleagues showed that supramammillary nucleus (SuM) axons densely innervate the supragranular layer of the dentate gyrus and form monosynaptic connections with both granule cells and GABAergic interneurons (Hashimotodani et al., 2018). Using viral optogenetics, transgenic mouse lines, slice electrophysiology, and pharmacology, they demonstrated that SuM afferents co-release glutamate and GABA onto these postsynaptic targets. In another study, Hirai and colleagues employed minimal stimulation, asynchronous release analysis, and anatomical mapping of excitatory and inhibitory specializations to support the existence of distinct glutamatergic and GABAergic vesicle populations within the same SuM axons (Hirai et al., 2024). Similarly, in septo-hippocampal cholinergic terminals, Takács and colleagues integrated optogenetics and slice electrophysiology with STORM imaging and electron tomography to demonstrate that acetylcholine and GABA are localized to separate vesicles and are regulated by different calcium channels (Takacs et al., 2018). Zych and Ford provide a complementary methodological example by directly comparing two co-transmitted signals from the same dopamine projection using simultaneous postsynaptic readouts, optogenetic activation, pharmacological dissection, dLight dopamine imaging, fast-scan cyclic voltammetry, calcium chelation, and conditional removal of RIM proteins from dopamine neurons. Together, these studies emphasize that demonstrating co-transmission requires more than showing that two transmitters are present or released from the same axonal population; it requires determining whether the two signals arise from shared or distinct vesicle pools and whether they follow the same release rules.

Another significant development is the introduction of genetically encoded neurotransmitter sensors, which enable direct monitoring of transmitter dynamics rather than relying solely on postsynaptic currents. GACh sensors have facilitated optical measurement of acetylcholine release both *in vitro* and *in vivo* (Jing et al., 2018), iGABASnFR extended this strategy to GABA (Kolb et al., 2026), and newer iGluSnFR variants improved the temporal resolution and sensitivity of glutamate imaging (Helassa et al., 2018; Aggarwal et al., 2023). Although these tools do not independently establish vesicle identity, they increasingly allow researchers to assess whether different transmitter components are recruited with distinct timing, probability, or state dependence in intact circuits. Nevertheless, notable limitations remain. Optical sensors detect only extracellular transients and do not reveal vesicle organization. Ultrastructural methods primarily provide static images, and minimal stimulation can be influenced by failures or incomplete bouton isolation. Continued progress in the field will likely depend on explicitly multimodal experiments that integrate projection-specific manipulation, quantal electrophysiology, nanoscale anatomy, and direct optical measurements of transmitter release.

## 6 Discussion

Accumulating evidence has revised the traditional one neuron–one transmitter framework by showing that neurotransmitter co-transmission is a more common and functionally important feature of neural circuits than previously appreciated. Current evidence indicates that many neurons utilize multiple transmitters through distinct organizational architectures, such as co-packaging within a single vesicle, release from parallel vesicle pools within the same terminal, or segregation across separate boutons or neuronal processes. These configurations are not merely structural variants of a single phenomenon. Rather, they establish distinct rules for neurotransmitter release, target engagement, and short-term synaptic dynamics, thereby influencing circuit computation in fundamentally different ways.

Several common principles of co-transmission have emerged across neural systems. Co-transmission broadens signaling across multiple timescales, enhances target specificity, and permits dynamic shifts in transmitter balance according to activity and circuit state. Thus, co-transmission functions as a mechanism for increasing the flexibility, precision, and context dependence of neural communication, rather than serving as a mere biochemical curiosity. However, the field is constrained by the challenge of inferring release mechanisms from any single experimental approach. Consequently, progress has relied on integrating molecular profiling, synaptic physiology, high-resolution anatomical studies, and, increasingly, direct optical measurements of transmitter release.

A significant challenge for future research is to establish direct links between synaptic architecture and function *in vivo*. The stability of specific forms of co-transmission as cellular traits, their potential for state-dependent reconfiguration, their modulation by development or experience, and their roles in disease-related circuit dysfunction remain unresolved. Addressing these issues will require multimodal approaches capable of resolving transmitter identity, vesicle organization, and release dynamics at the level of defined synapses and behaviorally relevant circuit states. As these methodologies advance, co-transmission is poised to become a central framework for understanding how neural circuits diversify their output and adapt signaling to meet changing physiological demands.
